# Encapsulation of Bioactive Peptides by Spray-Drying and Electrospraying

**DOI:** 10.3390/foods12102005

**Published:** 2023-05-15

**Authors:** Carmen Berraquero-García, Raúl Pérez-Gálvez, F. Javier Espejo-Carpio, Antonio Guadix, Emilia M. Guadix, Pedro J. García-Moreno

**Affiliations:** Department of Chemical Engineering, University of Granada, 18071 Granada, Spain; carbegar@ugr.es (C.B.-G.);

**Keywords:** nano/microencapsulation, spray-drying, electrospraying, coaxial encapsulation, bioactive peptides, protein hydrolysates

## Abstract

Bioactive peptides derived from enzymatic hydrolysis are gaining attention for the production of supplements, pharmaceutical compounds, and functional foods. However, their inclusion in oral delivery systems is constrained by their high susceptibility to degradation during human gastrointestinal digestion. Encapsulating techniques can be used to stabilize functional ingredients, helping to maintain their activity after processing, storage, and digestion, thus improving their bioaccessibility. Monoaxial spray-drying and electrospraying are common and economical techniques used for the encapsulation of nutrients and bioactive compounds in both the pharmaceutical and food industries. Although less studied, the coaxial configuration of both techniques could potentially improve the stabilization of protein-based bioactives via the formation of shell–core structures. This article reviews the application of these techniques, both monoaxial and coaxial configurations, for the encapsulation of bioactive peptides and protein hydrolysates, focusing on the factors affecting the properties of the encapsulates, such as the formulation of the feed solution, selection of carrier and solvent, as well as the processing conditions used. Furthermore, this review covers the release, retention of bioactivity, and stability of peptide-loaded encapsulates after processing and digestion.

## 1. Introduction

**Figure 1:**
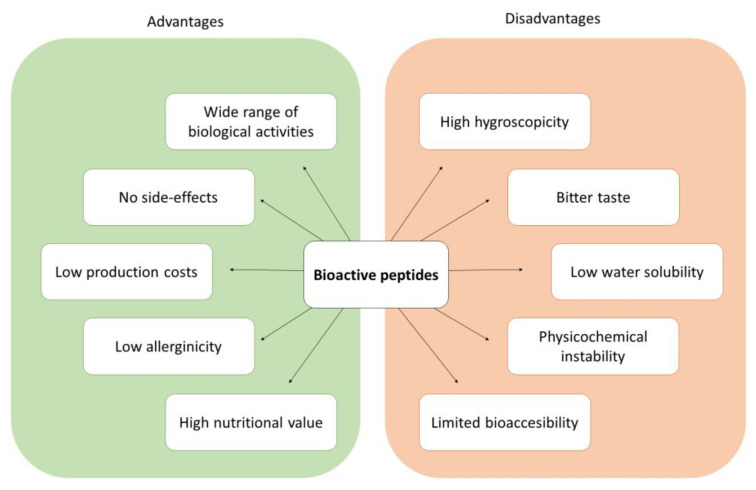
Advantages and disadvantages of using peptides as bioactive ingredients in food.

Bioactive peptides have received increasing interest in the last few decades due to the wide range of biological activities they can exert. Multiple studies have demonstrated their antioxidant, antihypertensive, antimicrobial or anti-inflammatory activities, among others, as well as their high potential for the treatment of various diseases, such as diabetes and different types of cancers [1]. This has boosted the research on the use of biopeptides as therapeutic agents, especially for the treatment of certain chronic conditions, through their incorporation in supplements, pharmaceutical compounds, or functional foods [2]. In addition to the bioactive properties previously mentioned, biopeptides have other advantages such as their low production cost, low allergenicity, high nutritional value and high digestibility (Figure 1) [3].

Enzymatic hydrolysis is commonly used to produce bioactive peptides [4]. This technology releases the peptides encrypted in the original protein by means of breaking the peptide bonds with proteases, improving the technological and bioactive properties of the hydrolysates, enhancing their digestibility, and decreasing their antigenicity by degrading the allergenic epitopes [5]. Bioactive peptides are usually of 2–20 amino acids in length, and their activity is determined by the specific amino acid sequence and the relative abundance of certain residues (e.g., hydrophilic, hydrophobic, or aromatic) within the peptide [6].

Clinical application of bioactive peptides is severely limited by the difficulty to find an effective method of administration [7]. In the pharmacological field, most protein-based treatments are administered via parenteral injection. However, this administration approach presents several drawbacks, mainly (i) difficulty in self-administration, (ii) short half-life of the proteins, and (iii) proteins can be easily degraded in the bloodstream [8]. Although oral administration of bioactive peptides is a much easier and more practical approach to implement for consumers, there are multiple challenges in its use that must be overcome (Figure 1):

1. High hygroscopicity, which may result in physicochemical instability and loss of bioactivity [9].
2. Bitterness due to the exposure to taste receptors of hydrophobic amino acid residues generated from hydrolysis. It has a negative impact on consumer’s acceptance [10].
3. Low water-solubility, limiting the introduction of hydrolysates or peptides into food matrices, which requires generating dispersed systems [11].
4. Physicochemical instability during storage, processing, and digestion, due to the exposure of peptides to environmental conditions (e.g., oxygen, heat) or their interaction with the digestive proteases and other compounds present in the food matrix [12].
5. Limited bioaccessibility. Once ingested, peptides must be able to remain intact until they are absorbed in the intestine in order to exert their bioactivity. This is challenging due to the harsh conditions found in the gastrointestinal tract, such as the strongly acidic pH in the stomach and the enzymatically active gastric and intestinal fluids [13].

To overcome these limitations, great efforts are focused on increasing the physicochemical stability and bioaccessibility of peptides. Indeed, stabilization of bioactive peptides is an essential process to ensure that their in vitro activity properly translates to in vivo animal and human models after processing, storage, and digestion [14]. Encapsulation of bioactive peptides, consisting in entrapping the peptides within a matrix or layer(s) of encapsulating agent(s), reduces hygroscopicity, masks bitterness and protects the biopeptides from degradation during processing, storage and digestion while maintaining their bioaccessibility [15]. The pharmaceutical industry has extensively used encapsulation techniques for the stabilization and controlled release of active compounds. Encapsulation processes have attracted much attention from the food industry, especially due to the growing interest during recent decades in the fortification of food with bioactive ingredients, including peptides, to produce functional food products [14]. Although significant research has been carried out on the encapsulation of lipids (e.g., omega-3 fatty acids), probiotics, vitamins and antioxidants (e.g., polyphenols), there are considerably fewer studies on the encapsulation of bioactive peptides [3,9,14].

Several techniques are available for the entrapment of food bioactives (i.e., peptides) within a biopolymer matrix (i.e., spray-drying, spray-cooling, fluid-bed coating, extrusion, electrospraying and complex coacervation followed by drying) [16]. Nevertheless, spray-drying is the most commonly used technique for the encapsulation of thermosensitive compounds, which permits obtaining dry microparticles at an industrial scale [17] without peptide degradation (e.g., changes in secondary structure) [18]. In recent years, electrospraying has emerged as a promising technique to encapsulate bioactive ingredients at room temperature, obtaining nano/microcapsules with narrow size distribution, low cost, and potential to be scaled up [19]. In any case, it should be noted that the potential degradation of peptides due to electrostatic stresses during electrospraying might need further investigation [18]. Furthermore, both spray-drying and electrospraying can work in coaxial configuration, resulting in capsules with a double layer of encapsulating wall(s), which might enhance the protection and delivery of the bioactives [20]. Contrarily to coaxial spray-drying and electrospraying, other encapsulating techniques such as fluidized-bed and spray-chilling processes require an additional production stage to provide a second coating of the encapsulates, which can result in double-layered capsules [21].

In the light of the above, this work presents a systematic review on the encapsulation of bioactive peptides and protein hydrolysates by spray-drying and electrospraying. This review focuses on the various factors affecting the properties of the encapsulates (e.g., morphology, size, encapsulation efficiency), such as the formulation of the feed solution including the type of carrier and solvent. The specific processing conditions used for both encapsulation techniques (i.e., inlet and outlet air temperature for spray-drying and voltage and flow rate for electrospraying, among others) were reviewed. The use of mono- or coaxial encapsulation methods for both spray-drying and electrospraying are discussed. Finally, this review focuses on release, bioactivity and stability after digestion of the encapsulated peptides.

## 2. Literature Search

A literature search of the papers reporting the encapsulation of peptides, protein hydrolysates and proteins via spray-drying and electrospraying, both monoaxial and coaxial, published in the period between 2012 and November 2022 was carried out using Scopus (https://www.scopus.com/(accessed on 25 November 2022)). Research papers containing the keywords “peptide & encapsulation & spray drying,” “hydrolysate & encapsulation & spray drying,” “protein & encapsulation & spray drying,” “peptide & encapsulation & electrospraying,” “hydrolysate & encapsulation & electrospraying,” “protein & encapsulation & electrospraying,” “coaxial & encapsulation & electrospraying” and “coaxial & encapsulation & spray drying” were searched. Consequently, the literature search yielded 444 references that were manually screened. From all the works, 397 were excluded because: (a) the paper was published before 2012 (*n* = 87), (b) the reference was not a research paper (*n* = 71), (c) the reference was repeated in different searches (*n* = 105), (d) peptides, protein hydrolysates or protein were not the active ingredient encapsulated (*n* = 71), (e) spray-drying or electrospraying was not used to dry the formulations (*n*  = 46), and (f) no information about the spray-drying process or formulation was available (*n*  =  17). Additionally, three works not found in the Scopus literature research were deemed adequate to be added. A total of 50 experimental research papers were considered for the evaluation.

## 3. Encapsulation of Protein-Based Bioactives by Spray-Drying

### 3.1. Fundamentals of Spray-Drying

**Figure 2:**
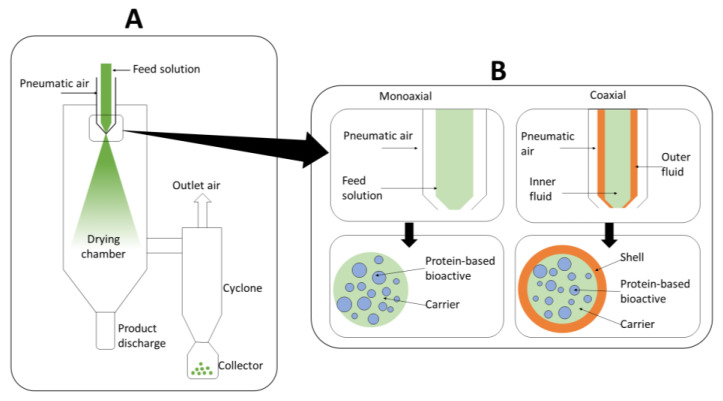
(**A**) Scheme of spray-drying process. (**B**) Nozzle configuration and cross section of the microcapsules obtained for monoaxial and coaxial spray-drying for the encapsulation of protein-based bioactives.

Spray-drying is an encapsulation technique based on the atomization of a solution into droplets and their subsequent drying within a gas (e.g., air or nitrogen) at high temperature, producing dry particles [22]. More specifically, the encapsulation of bioactives by spray-drying consists in the dispersion/mixing of the bioactive together with a carrier (encapsulating agent) in the selected solvent. The solution is pumped and atomized at the entrance of the drying chamber using an atomizer. The atomizer type (i.e., rotary atomizer, pressure nozzle, or sonic nozzle) is selected depending on the characteristics and desired particle size of the final product. The pressure nozzle is the most used at laboratory scale (Figure 2A) [23]. A gas stream at high temperature is concurrently fed to the chamber, providing the driving force to the drying process (e.g., difference in temperature and relative humidity between the droplet and the inlet air). Most of the water is removed during the first drying stage, where the droplet surface is saturated with water. The evaporation of the solvent provides the cooling needed to maintain the surface temperature at a constant value (i.e., wet-bulb temperature). The second drying stage, known as the falling rate period, begins when the surface of the droplet is no longer saturated with water, resulting in the formation of a thin crust. This crust limits water diffusion to the surface, reducing the evaporation rate and causing an increase in the temperature of the dried particle. The dry particles are separated from the outlet drying gas in a cyclone (Figure 2A) [23].

Contrarily to other drying processes (e.g., freeze-drying), spray-drying is normally operated in continuous mode at industrial scale, resulting in high encapsulation efficiency (EE) and minimal degradation of thermolabile bioactive ingredients [24,25]. During spray-drying, multiple parameters must be optimized to achieve high encapsulation efficiency and reduced heat damage. The parameters to be taken into account are based on the formulation of the solution/dispersion/emulsions, i.e., the carrier type, the ratio between the mass of peptide and that of the carrier (core:wall), and the pretreatments needed, such as the formation of nanoliposomes or nanoemulsions [26,27]. Drying kinetics are governed by heat and mass transport. Processing variables of the drying process influencing these transport mechanisms are temperature and humidity of the inlet gas, feed flow rate or flow rate of the drying gas [23,28].

- Inlet gas temperature is a key parameter that provides the driving force for the solvent evaporation. The temperature should be set at a level that is sufficiently high to promote water evaporation and ensure complete drying, without leading to agglomeration or deposition of wet particles on the chamber wall. Additionally, an increase in the temperature of the inlet air leads to a decrease in its relative humidity, which promotes water transport. However, excessively high inlet temperatures can lead to prompt crust formation, which limits water diffusion and subsequent evaporation. Therefore, careful control of the temperature of the inlet gas is necessary to ensure effective microencapsulation [29].
- Feed flow rate determines the size of the atomized droplets as well as the amount of water to be evaporated, which influences the temperature of the outlet gas and of the resulting particles.
- Drying-gas flow rate determines the amount of water evaporated and the residence time of the particles in the drying chamber. Too low a flow rate results in higher water condensation, as well as agglomeration or deposition of particles in the drying chamber. On the other hand, too high a flow rate could lead to degradation of the particles by shear stress.

The type of nozzle employed for atomization allows us to process: (1) only one liquid solution in a two-fluid nozzle, known as monoaxial spray-drying, or (2) two concentric liquid solutions in a three-fluid nozzle, known as coaxial spray-drying (Figure 2B). The type of process carried out (e.g., mono- or coaxial) affects the distribution of the bioactive compound within the matrix of encapsulating agent(s) (Figure 2B). For instance, in monoaxial spray-drying, the bioactive is dispersed within the carrier matrix. On the contrary, the encapsulation by coaxial spray-drying forms two layers of encapsulating agent(s), making it possible to disperse the bioactive within the core matrix of the carrier and forming an additional layer of encapsulating agent (Figure 2B). Nevertheless, it should be noted that both monoaxial and coaxial spray drying have some disadvantages, such as the wide size range (5–100 µm) and particle size distribution of the obtained powder. The latter might lead to capsules with different performance in terms of protection and delivery of the bioactive ingredient [23,30].

### 3.2. Encapsulation by Monoaxial Spray-Drying

#### 3.2.1. Formulation of the Feed Stream

Different methods are currently available to formulate the feed stream containing both the bioactive and the encapsulating agent. Blending is commonly reported in the literature, with 20 of the 29 papers found (69%) using it (Table 1). This method consists in mixing/dispersing the bioactive protein or peptide with the selected carrier, typically protein- or polysaccharide-based, in a selected solvent.

The use of proteins as carriers is widely used for the encapsulation of other bioactive compounds, such as fatty acids or vitamins [62,63,64,65], due to their functional properties, such as emulsifying, water holding, gelling, and film-forming capacities [26]. However, their use for the encapsulation of protein-based bioactives is still very limited, being reported in only two of the articles found in the literature (Table 1). This is mainly explained by the fact that the use of a carrier with a biochemical composition considerably similar to the bioactive compound to be encapsulated can lead to limitations in stability or expected bioactivity [66]. Among the few studies using proteins as carrier, Wang et al. [45] used soy protein isolate and maltodextrin (MD) (1:1) for the encapsulation of antioxidant soy protein hydrolysates at two different core:wall ratios (1.2:1 and 0.8:1). Similarly, Wang et al. used rapeseed protein isolates modified by acylation and high pressure for the encapsulation of rapeseed peptides [57,58] (Table 1). These studies reported particle sizes and encapsulation efficiencies consistent with those achieved using different carriers (Table 1).

Polysaccharides are the most used carriers for encapsulation of protein-based bioactives by spray-drying using a blend feed (Table 1). These biopolymers are abundant and inexpensive, as well as chemically stable [67]. Maltodextrin (MD), a derivative from starch with a dextrose equivalent ranging from 3 to 20, is commonly cited in the literature for the encapsulation of both protein hydrolysates [34,35,36,37,48] and peptides [53,59] (Table 1). MD shows good water solubility, high glass transition temperature and no flavor or odor [68]. Salleh et al. [33] studied the use of MD for the encapsulation of edible bird’s nest hydrolysates, as well as its combined use with other polysaccharide-based carriers such as carboxymethyl cellulose (CMC) and xanthan gum (XG), demonstrating that the encapsulates produced with MD-XG combination exhibited the best characteristics, with lower water activity, high solubility, and highest retention of the bioactivity.

Another polysaccharide-based carrier found in the literature is chitosan (CS) (Table 1). CS is characterized by being nontoxic, biocompatible, and biodegradable [67], which makes it very interesting as biomaterials for protein encapsulation. In addition, its mucoadhesive and intestinal epithelium-penetrating properties make it an ideal carrier for the oral delivery of proteins [67]. Despite these advantages, its use is notably limited. Aquino et al. [46] combined CS and mannitol for the oral delivery of spirulina bioactive peptide extracts. Gómez-Mascaraque et al. [49] compared the effect of encapsulating whey protein hydrolysate with both gelatin and CS, determining that CS was more effective at stabilizing the peptide while not affecting the profile of the peptides after digestion.

An alternative approach for the generation of the feed solution involves the development of nanoliposomes. Nanoliposomes are lipid-based systems composed of a single or multiple concentric bilayers made of phospholipids constituting a vesicle, which allows the storage of the bioactive peptides both in the aqueous core or in the interior of the bilayer [69]. Sarabandi et al. [38] studied the encapsulation of flaxseed protein hydrolysates dissolved in a phosphate-buffered solution (PBS) via nanoliposome formation using cholesterol, as well as lecithin and Tween 80 as surfactants. A MD solution was added to the nanoliposomes in a 1:1 (*v*/*v*) ratio before spray-drying. The effect of CS coating of the flaxseed protein hydrolysate–MD nanoliposomes was further studied, showing improvement in the physical properties of the particles (e.g., smaller particles after reconstitution, improved solubility, higher encapsulation efficiency) [39]. Mohammadi et al. [47] studied the effect of adding a CS coating to encapsulated spirulina platensis nanoliposomes, finding that it effectively improved the physical stability of the vesicles during storage by reducing particle aggregation. Similarly to nanoliposomes, nanoemulsions also use lipidic carriers. Oil-in-water nanoemulsions are colloidal systems with a hydrophobic liquid core composed of the oily/organic phase dispersed in the aqueous phase [70]. De Figueiredo Furtado et al. [51] produced single emulsions dispersing whey protein isolate and a mix of MD and lactose in an aqueous phase, while the oily phase was composed of a blend of high oleic sunflower oil, coconut oil and caprylic/capric triglyceride. They concluded that using oil blends with medium-chain triacylglycerols favors the formation of smaller spray-dried particles. Thus, the use of chitosan coating and medium-chain triglycerides can improve the physical properties of the particles. Zhu et al. [54] obtained emulsified droplets loaded with exenatide, which is a synthetic incretin used in the treatment of diabetes mellitus type 2. In this case, the protein-based bioactive was dispersed in the aqueous phase and poly(lactic-co-glycolic acid) (PLGA) in the organic phase composed of dichloromethane (DCM) and dimethyl carbonate (DMC) (1:1), resulting in particles with higher release and degradation compared to an alternative encapsulation method, such as ultrafine particle processing, which is based on disk rotation principles.

Double-emulsion systems have also been developed for the encapsulation of protein-based bioactives by monoaxial spray-drying. Ying et al. [59] produced a double emulsion (W1/O/W2) of soy peptides using different emulsifiers (i.e., polyglycerol polyricinoleate, lecithin and Span 60). To this end, a primary (W1/O) emulsion was obtained combining the soy peptides and different emulsifiers in the aqueous phase, whereas medium-chain triglycerides composed the oily phase. This primary emulsion was mixed with the W2 phase, which contained OSA modified starch and MD. The final double emulsion was spray-dried to produce peptide-loaded microcapsules; however, the authors reported that during drying, the double emulsion was ruptured, resulting in low encapsulation efficiency. Calderón-Oliver et al. [56] compared the use of blend and emulsion methods for the encapsulation of nisin, an antimicrobial peptide, using either pectin or alginate as carrier. They produced a double W1/O/W2 emulsion by combining the aqueous phase (nisin and avocado peel extract as an antioxidant) and the oily phase (soybean oil with monoglycerides as emulsifier) to produce the primary emulsion (W1/O). The final W1/O/W2 was obtained by mixing the primary emulsion with a collagen solution and the carrier (either pectin or alginate) solution. The emulsion method resulted in improved encapsulation efficiency.

In summary, when it comes to selecting the optimal formulation method for the encapsulation of bioactive peptides by spray-drying, it is important to consider the advantages and disadvantages of each approach. While feeds obtained by blending the peptides and carriers are simply prepared, leading to high encapsulation efficiency (Table 1), preparation of emulsion-based feeds can lead to degradation of the peptides due to their exposure to oil–water interfaces and the mechanical stress to which they are subjected during the emulsification process [46]. Moreover, the production of nanoliposomes has the disadvantage that the particles can suffer lipid oxidation during production and storage, limiting their shelf life [14]. Therefore, careful consideration should be given to the specific properties of the bioactive peptides and the intended application of the encapsulated product when selecting a formulation method.

In addition to the method of preparation of the feed solution and the type of carrier used, there are other characteristics of the solution that affect the encapsulation process, as well as the morphology and release of the capsules produced. The peptide:carrier or core:wall ratios greatly affect the encapsulation efficiency, which decreases when increasing the load of peptide [14]. Akbarbaglu et al. [37] studied the effect that core:wall ratios of 1:1, 1:2 and 1:3 (*w*/*w*) had on the encapsulation of flaxseed protein hydrolysates using MD as carrier. They found that increasing the concentration of carrier resulted in a higher production yield, while the moisture content and water activity decreased. Likewise, Palamutoğlu and Sariçoban [34] encapsulated fish collagen hydrolysates with MD at 1:4 and 1:9 (*w*/*w*) core:wall ratios and found that moisture content and water activity decreased with increasing concentration of MD. Similar results were also obtained by encapsulation of rapeseed peptides using rapeseed protein isolates as wall materials at different core:wall ratios (1:1, 1:2 and 2:1 *w*/*w*), and the production yield increased when more carrier was used [58]. Taken together, the core:wall ratio is an important factor that affects the encapsulation efficiency of bioactive peptides. Increasing the concentration of carrier can improve the production yield while reducing the moisture content and water activity of the encapsulates, leading to better entrapment of the bioactive peptides.

#### 3.2.2. Processing Conditions

Apart from the formulation of the feed stream, the processing conditions also influence the properties of the encapsulates obtained. The inlet temperature of the drying gas is a key variable determining the drying kinetics. The works reported in the literature on the encapsulation of protein-based bioactives use temperature values in the range of 60–200 °C (Table 1). The lowest inlet temperature was employed by Zhu et al. [54], who carried out the encapsulation of exenatide by spray-drying using inlet air at 60 °C. This low temperature, out of the norm for spray-drying, was due to the use of organic solvents (DMC and DCM) instead of water, as these evaporate at lower temperatures. The highest inlet temperature was 200 °C, which was used for the encapsulation of whey protein hydrolysate [50] and glutathione [55] employing β-cyclodextrin (β-CD) or CS as encapsulating agents (Table 1). Yang et al. [50] reported outlet temperatures of 90 ± 5 °C, while Webber et al. [55] reported 72 °C. This difference could be due to the joint effect of other parameters such as feed flow rate, drying air flow rate and solid concentration of the feed solution. Cao et al. [44] studied the effect of inlet temperature on the encapsulation of watermelon seed hydrolyzed protein with MD. They compared inlet temperatures of 150, 160, 170, and 180 °C, finding that increasing temperature led to lower moisture content and solubility of the capsules. Likewise, particle size slightly increased with increasing temperature, varying from an average particle diameter of 10 µm at 150–160 °C to 12.9 µm at 170 °C. These results agree with a previous study [71] on the encapsulation of whey protein concentrates, reporting larger particles with increasing inlet temperature. This was attributed to elevated inlet temperatures increasing the moisture removal rate, forming a crust more quickly and limiting the shrinking time of the particle. Thus, the inlet temperature must be selected based on the nature of the solvent and the bioactive to be encapsulated, as well as other parameters like feed flow rate, drying air flow rate, and solid concentration of the feed solution. While high inlet temperatures increase the moisture removal rate, which can lead rapid crust formation and then larger particles, it also increases the risk of thermal degradation of bioactives. Therefore, it seems reasonable to use inlet temperatures between 130 and 190 °C and to avoid outlet temperatures above 100 °C to obtain encapsulates with minimal thermal degradation.

Feed flow rates reported in the literature range from 160 to 1380 mL/h (Table 1). This variable affects the size of the atomized droplets and consequently the size of the dried particles [72]. The lowest feed flow rate (160 mL/h) value was applied to encapsulate buffalo whey protein hydrolysates, with gum arabic (GA) and MD as encapsulating agents, obtaining particles of average diameter between 2 and 20 μm when using a two-fluid nozzle with 700 μm inner diameter [31]. The highest feed flow rate was 1380 mL/h and was used for the encapsulation of *P. lunatus* hydrolysates with GA and MD, forming particles with diameter of 3.3–6.8 μm when using a two-fluid nozzle for atomization with diameter of 500 μm [42]. Although it is accepted that a higher feed flow rate results in larger particles due to collision and subsequent fusion of small drops [73], the combined effect of the other processing variables could justify that the particles obtained in the latter study being smaller. Although high feed flow rates are desired for higher production rates, they can also result in condensation on the walls or wet particles in the chamber or cyclone due to higher humidity increasing stickiness and agglomeration, leading to a decrease in particle recovery attributed to wall deposition [60]. Thus, ratios of 300–500 mL/h are the most preferred. Curiously, there was a wide difference between the pneumatic air pressures used (40 and 1 bar), which has been proven to affect the particle size during the atomization process [73].

Regarding the correlation between the nozzle diameter and the morphology of the particles obtained, Keogh et al. [74] studied the effect of the nozzle diameter in the processing of milk powders and observed that the particle diameter of the spray-dried powders increased when increasing the nozzle diameter. However, the literature in this regard is very limited. Only two nozzle sizes were used in the literature found (500 and 700 µm), making it not possible to establish a correlation between these values and the particle size results.

It is noteworthy that both the solubility and shelf stability of the powdered product is affected by the physicochemical properties of the encapsulates, such as morphology, particle size distribution, moisture content, or encapsulation efficiency. Smallness (under 5 µm) and a tight distribution of particles is desired to improve shelf stability [28]. Regarding the morphology of the particles, it was found that the size of the encapsulates produced by monoaxial spray-drying ranged from 0.132 μm [39] to 183 μm [35], with 21 of 24 articles reporting diameter values from 0.25 to 50 μm. These results are consistent with previous studies reporting typical dry particle diameter in laboratory scale spray-dryers of 0.5 to 50 μm [75]. It is also noteworthy that four of the five studies reporting average size below 1 μm were obtained via formation of nanoliposomes and similar conditions were used for three of the works found, with inlet air temperature of 130–170 °C and feed flow rate of 300 mL/h [38,39,41].

Encapsulation efficiency (EE) is one of the main parameters determining shelf-life stability. EE can be defined as the percentage of bioactive compound, whether peptide, hydrolysate, or protein, that is trapped inside the carrier matrix with respect to the amount initially added. Lower EE values lead to more exposed bioactive on the surface of the capsules, which is more easily degraded, resulting in reduced bioactivity [14]. All studies reported in the literature on the encapsulation of protein-based bioactives by monoaxial spray-drying obtained EE values over 50%, except for Ying et al. [59], who reported EE values in the range 29.51–45.83% for the encapsulation of soy peptides via W1/O/W2 because the emulsion was not physically stable (Table 1). The highest EE (~100%) was reached by Koker et al. [60], where ovalbumin was encapsulated using dextran sulfate and poly-l-arginine as carrier for the elaboration of vaccine antigens. EE of peptide-loaded encapsulates can be improved by the addition of surfactants. For instance, Tween 80 was used to reduce flaxseed peptide migration to the air–water interface in spray-drying when using MD as carrier [36]. The authors reported reduced degradation by shear stress and dehydration, leading as well to higher bioactivity (i.e., antioxidant activity), indicating that the addition of surfactants should be considered in the future to improve encapsulation of peptides by spray-drying.

Altogether, processing conditions play a significant role in determining the properties of the encapsulates obtained. The inlet temperature of the drying gas is a crucial variable that affects drying kinetics, particle size, and morphology. Inlet temperatures between 130 and 190 °C are found to obtain desirable encapsulates with minimal thermal degradation. Feed flow rate also affects the size of the atomized droplets, and consequently the size of the dried particles. It was observed that particle size under 5 µm and a narrow distribution of particles improve shelf stability of the encapsulates. Finally, feed flow rate ratios of 300–500 mL/h are preferred to avoid the formation of wet particles or wall deposition in lab spray-dryers.

### 3.3. Encapsulation by Coaxial Spray-Drying

Lately, several works have been reported on the coaxial encapsulation of food bioactives by spray-drying using a three-fluid nozzle. This configuration (Figure 2B) allows feeding two different solutions through two concentric channels in the nozzle while the pneumatic air responsible for the atomization flows in the external channel [76]. In this way, the bioactive compound dissolved in a solution of carrier (core solution) can be pumped through the inner channel, and a solution containing the same or a different carrier (shell solution) is pumped through the outer channel. Both the shell and core solutions meet concentrically at the tip of the nozzle being atomized and dried in the chamber to form microcapsules with a core–shell structure. Therefore, this method might offer greater protection and better control for the release of the bioactive compound when compared to the monoaxial process (two-fluid nozzle) [77].

Despite the advantages of this method and its widespread use in the encapsulation of bioactive compounds such as carotenoids, triglycerides or organosulfur compounds [78,79,80], the literature on the encapsulation of bioactive protein hydrolysates and peptides by coaxial spray-drying is very limited. To the authors’ knowledge, only one paper has been published regarding the production of protein-loaded microparticles via spray-drying using a three-fluid nozzle [20]. In this work, the authors encapsulated lysozymes employing an aqueous solution of trehalose as core solution and a shell solution consisting in PLGA dissolved in a mixture of solvents (acetonitrile, DCM, and acetone). The effect of the core:shell flow rate ratio (4:1 or 10:1) was studied. The operating conditions selected were (i) inlet temperature = 60 °C, (ii) outlet temperature = 40–45 °C, and (iii) drying air flow rate = 37500 L/h. The study concluded that solvent selection did not affect particle size, while it did affect EE. On the other hand, the feed flow rate ratio did affect the particle size, which ranged from 1.07 to 1.60 μm. Hence, the coaxial encapsulation by spray-drying of bioactive protein hydrolysates and peptides deserves further research.

## 4. Encapsulation of Protein-Based Bioactives by Electrospraying

### 4.1. Fundamentals of Electrospraying

Electrospraying or electrohydrodynamic atomization is a drying and encapsulation technique based on the application of an electric field to a solution to obtain dried nano/microstructures at room temperature [81].

**Figure 3:**
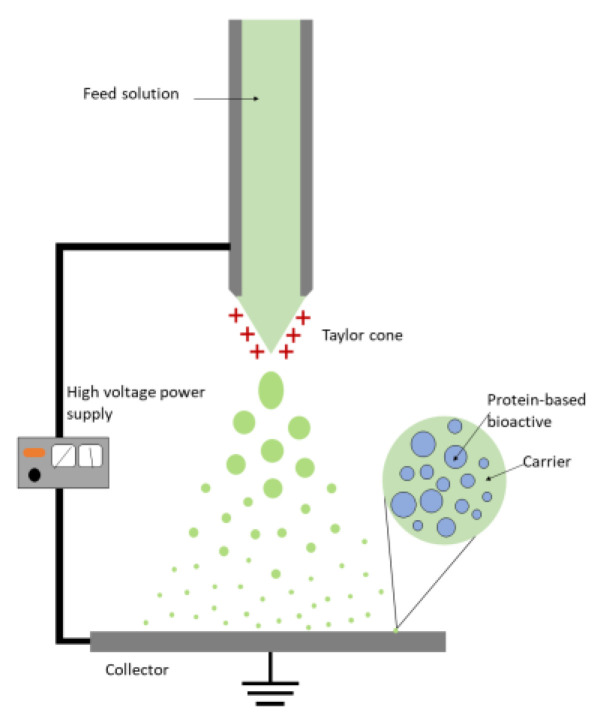
Scheme of monoaxial electrospraying process and theoretical cross section of the nano/microcapsules obtained.

Electrospraying consists of pumping a solution, dispersion, or emulsion that contains the protein-based bioactive through a capillary injector/needle of a conductive material [82]. A grounded collector is placed opposite the needle at a given distance, and an electric field is applied between the injector and the collector. The solution is pumped through the needle at a regulated flow rate, and if no voltage is applied, as the drop of solution emerges from the needle a meniscus is formed. When the electrostatic field is sufficiently high, the air–liquid interface of the meniscus is polarized, causing it to deform into a conical shape, known as a Taylor cone [81]. As the voltage continues to increase, it reaches a point at which the surface tension is no longer able to hold the liquid in the droplet, resulting in the emission of a jet from the tip of the cone directed towards the collector. The jet breaks into a spray of charged particles due to the low viscoelasticity of the solution and the electrostatic repulsion forces that take place. In the travel of the droplets towards the collector, the solvent(s) used is evaporated and dry nano/microparticles are obtained (Figure 3) [83].

Depending on the properties of the solution and the processing parameters used, two different methodologies can be applied, mainly (i) electrospraying, where the intermolecular cohesion of the fluid is low enough that the electrostatic forces break the jet emitted from the solution into small droplets that result in the formation of nano/microparticles after solvent evaporation, or (ii) electrospinning, where the high molecular cohesion avoids jet fragmentation, and after the evaporation of the solvent, it gives rise to the formation of ultrafine fibers [82]. Electrospraying is the preferred process for obtaining food ingredients, since electrospun fibers, contrarily to nano/microcapsules, result in continuous mats that are difficult to disperse in any food matrix without prior breakage [19].

**Figure 4:**
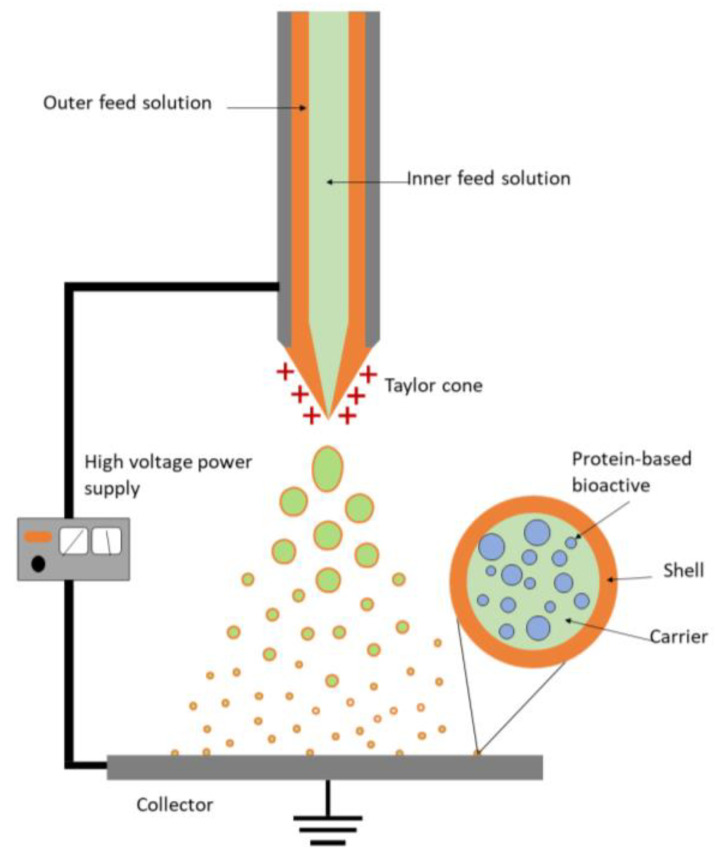
Scheme of coaxial electrospraying process and theoretical cross section of the nano/microcapsules obtained.

Electrospraying, as spray-drying, also allows working in both mono- and coaxial configurations. Monoaxial electrospraying typically results in the formation of amorphous solid dispersions containing the protein-based bioactives dispersed within the carrier matrix (Figure 3). Alternatively, coaxial electrospraying is a customized version of electrospraying, in which two different liquids are separately delivered through individual coaxial capillary needles (Figure 4). The solution containing the bioactive compound and potentially the encapsulating agent is pumped through the inner needle (core), while a second solution containing the same or a different encapsulating agent is delivered through an outer concentrical needle (shell) [84]. Therefore, a concentric Taylor cone of both solutions is formed at the tip of the needles, and when the solution and processing parameters are appropriately selected, it results in the formation of nano/microcapsules with a core–shell structure [85,86] (Figure 4). Coaxial electrospraying combines the advantages of monoaxial electrospraying, adding the ability to precisely control the core–shell shape, as well as better protecting the bioactive peptides from process-induced denaturation and aggregation [87].

Electrospraying parameters such as solution properties, processing variables and environmental conditions can affect the morphology, particle size and EE [88]. A high concentration of encapsulating agent leading to high viscosity and density of the solution can derive in the formation of larger particles, while increasing electrical conductivity of the solution results in particles with smaller diameter. Regarding the processing variables, a high electric potential between injector and collector results in smaller particle diameter, whereas increasing solution flow rate increases particle size. Long injector–collector distances allow for better evaporation of the solvent, while short distances may result in wet and collapsed particles. Environmental conditions such as temperature and humidity also affect the drying kinetics, since they determine the driving forces for the drying process. In addition, other factors should be considered when encapsulating protein-based bioactives by electrospraying: (1) proteins lead to highly conductive solutions that prevent charge formation, which reduces the stability of the Taylor cone, and (2) the use of food-grade solvents such as water leads to solutions with high surface tension that hinder jetting [86].

Electrospraying encapsulation has been widely used in the pharmacological field due to its low cost, easy operation and improved bioaccessibility of the nano/microcapsules obtained [8]. However, its use in food applications is still limited due to its low production capacity. To solve this limitation, several modifications have been reported, including: (i) multineedle electrospraying systems [89], (ii) free surface electrospraying systems [90], or (iii) pressurized-gas-assisted electrospraying [91]. However, this absence of application to the food industry is reflected in a lack of literature on the subject. The available information is mainly oriented to oral pharmacological supplementation, and no data were found on bioactive protein hydrolysates. Thus, further research should be carried out on the application of electrospraying encapsulation in foods.

### 4.2. Encapsulation by Monoaxial Electrospraying

#### 4.2.1. Formulation of the Feed Stream

The encapsulation of protein-based bioactives by monoaxial electrospraying requires the drying of only one solution containing the bioactive. The most common method to produce this feed stream is blending (e.g., dissolving the bioactive in a solution containing the carrier). Nine of the eleven works found in the literature used this approach (Table 2). Bock et al. [92] encapsulated bovine serum albumin (BSA) by electrospraying a blend feed stream where BSA was dissolved in chloroform or DCM using poly(ethylene glycol) (PEG) and poly(ε-caprolactone)(PCL)/PLGA as carrier. Similarly, Musaei et al. [93] prepared a blend feed stream using an ethanol–acetic acid mixture to encapsulate BSA using PLGA as encapsulating agent. Blend electrospraying has also been used to encapsulate larger molecules, such as the hormone angiotensin II using N-octyl-O-sulfate chitosan (NOSC) as a carrier [94], or the enzymes alkaline phosphatase with poly(ethylene oxide) (PEO) [95] and streptokinase with PLGA [96].

Although electrospraying is carried out at room temperature, which avoids thermal degradation of thermosensitive ingredients, the use of specific solvents may induce protein denaturation and loss of activity when exposure is prolonged [8]. Hence, an alternative approach to a blend for producing the feed stream is to obtain emulsions that prevent contact between specific solvents and the bioactives [8]. According to previous studies, encapsulation by emulsion electrospraying allows the formation of particles with core–shell structures similar to those that could be obtained by coaxial electrospraying [103]. This process is often used to mix two immiscible fluids, typically through a single W/O or double W1/O/W2 emulsion [8]. The two articles found in the literature using emulsion electrospraying were based on the drying of water-in-water (W/W) emulsions. Yao et al. [99] used this approach to encapsulate BSA in PLGA. For that, two immiscible solutions were prepared: the organic phase was composed of PLGA in chloroform and the aqueous phase was composed of the BSA dissolved in water. Similarly, Y. Song et al. [102] produced a W/W emulsion by dissolving β-amylase in the aqueous phase composed of dextran and sodium alginate, which was electrosprayed into a water solution containing CaCl_2_ and PEG, forming a calcium alginate shell containing the amylase core. As mentioned in Section 3.2.1, it should be noted that emulsion feed preparation is less used, as it presents difficulties in producing stable emulsions and the shear stress of mechanical mixing required for emulsion preparation could modify the protein-based bioactives [8].

The type of carrier and solvent used determine the main properties of the feed stream influencing the electrospraying process, such as viscoelasticity, conductivity, and surface tension [86]. A wide variety of natural and synthetic polymers are used as encapsulating agents in electrospraying, including biocompatible and biodegradable polymers such as gelatin, MD, pullulan, glucose syrup, dextran, hyaluronan, CS, PCL, poly(lactic acid) (PLA), PEG, PLGA, alginate, PEO, and NOSC, among many others. Carriers commonly used were alginate, PEO and NOSC, all of which are particularly used for the formulation of oral delivery drugs since they are all safe and present high biocompatibility. Alginate was used to encapsulate α-calcitonin gene-related peptide (α-CGRP) [97] and BSA/porcine interleukin-1β (pIL-1β) [100], both by blend electrospraying and resulting in particles with widely differing sizes, ranging from 194.23 ± 10.08 to 20 μm, respectively. PEO is a synthetic semicrystalline polymer mostly used for electrospinning due to its rheological characteristics, and thus only one work used it for electrospraying the enzyme alkaline phosphatase [95,104]. Likewise, NOSC was only used for the encapsulation of the hormone angiotensin II [94]. The most reported polymer carrier in the literature for the encapsulation of protein-based bioactives was PLGA (Table 2), a US Food and Drug Administration (FDA)-approved biocompatible copolymer that has been extensively used in biomedical devices with excellent application records in vivo [99]. Interestingly, Musaei et al. [93] found that increasing PLGA concentration did indeed affect the particle size of the capsules, increasing the size of the nanocapsules from 120 nm to 225 nm, which is related to increasing EE. Although all these biopolymers have shown good encapsulating capacity, studies have focused on drug release formulation and research on food application is very limited. Only PLGA has been studied for application in food fortification, with good results [105]. Polysaccharide- and protein-based carriers are commonly employed as encapsulating agents for encapsulation of protein-based bioactives by spray-drying; however their use for the encapsulation of these bioactives by electrospraying was not reported in the literature. These kinds of carriers are especially suitable for the food industry since they are food-grade and soluble in water, which avoids the use of non-food-grade solvents [105]. Therefore, further research on the use of food-grade, low-cost biopolymers for the encapsulation of protein-based bioactives by electrospraying is required.

#### 4.2.2. Processing Conditions

Processing variables (voltage, injector-to-collector distance and feed flow rate), together with feed solution properties, affect the characteristics of the nano/microcapsules obtained (e.g., morphology, size). The applied voltage for all the studies reported in the literature was kept between 2 and 20 kV (Table 2). The effect of voltage was assessed for the encapsulation of BSA by electrospraying using PLGA as carrier [93]. The authors compared three different voltages (10, 15 and 20 kV) and found that increasing voltage from 10 kV to 20 kV resulted in decreasing average diameter from 0.185 µm to 0.085 µm, which is desired to increase surface area and thus improve solubility and permeability. This effect was also found in electrosprayed amylase particles with PEG and dextran as carrier, where the applied voltages were adjusted from 2.6 kV to 2.85 kV to produce particles of different diameters [102]. However, applied voltages over 20 kV were found to alter protein-based bioactives. For instance, a study on the encapsulation of angiotensin II by electrospraying using NOSC as encapsulating agent showed that its stability was significantly reduced at 20 kV. Since the electric field strength is determined by both applied voltage and distance between nozzle and collector (N-C), changes in both parameters affect the stability of the bioactive compounds during processing.

Increasing feed flow rate is desired to increase productivity; however, it is linked to higher particle diameter. Low feed flow rate results in better encapsulation as well, and thus a compromise between productivity and quality of the capsules must be reached. Onyekuru et al. [95] studied the effect of feed flow rates ranging from 0.3 to 1.5 mL/h on the encapsulation of alkaline phosphatase with PEO, determining that although low flow rates produced better encapsulation, the optimum flow rate was 0.6 mL/h. Different feed flow rates were also compared for the encapsulation of serum albumin (SA) by electrospraying using PEG and PCL/PLGA as encapsulating agents [92]. It was reported that increasing the feed flow rate from 0.5 mL/h to 1 mL/h resulted in average diameters increasing from 5.6 ± 0.8 μm to 7.1 ± 1.7 μm. However, higher flow rates also resulted in uneven spread of the solution at the nozzle and an uncontrolled electrospraying of large droplets. This work also utilized different nozzle diameters (450–800 μm), but no effect on particle size was reported. On the other hand, Y. Song et al. [102] determined that the size of the particles could be reduced by using nozzles with a smaller diameter after comparing three different diameters (40, 170 and 320 μm). The consensus seems to be working at a low feed flow rate, but process productivity must not be compromised to be cost-effective.

The studies shown in Table 2 reported EE values for the nano/microcapsules loaded with protein-based bioactives ranging from 20% to 92%. The lowest EE was obtained for encapsulation of SA using PEG and PCL/PLGA as carriers [92]. Since higher EE has been linked to larger particles and lower protein loading [106], these parameters were studied. Indeed, the authors confirmed that increased particle diameter corresponded with higher EE values, with a critical size allowing optimum encapsulation. In the same study, lower protein loading also resulted in improved EE, but the extraction method used to measure EE presented limitations due to protein aggregation and the lack of use of surfactants. The highest EE was achieved by encapsulating BSA by emulsion electrospraying using PLGA as encapsulating agent [99]. This work showed that increasing the aqueous phase volume ratio (e.g., increasing bioactive load) resulted in decreased EE, varying from 92% at 5 µL/mL to 80% at 100 µL/mL. It was explained as being due to increased density of emulsion droplets in the feed solution, and thus increasing migration of the aqueous phase containing the BSA to the surface of the particle.

### 4.3. Encapsulation by Coaxial Electrospraying

The literature found regarding the encapsulation by coaxial electrospraying of protein-based bioactives was focused exclusively on the pharmacological/medical field. No works on the encapsulation by coaxial electrospraying of bioactive protein hydrolysates or peptides have been reported in the literature.

Only four works studying the coaxial electrospraying of proteins were found (Table 3). None of them used carrier in the formulation of the inner solution (core). For the outer solution (shell) PLGA was the most used encapsulating agent, appearing in two studies. This follows the trend established in monoaxial electrospraying, since, as previously mentioned, all the literature found was mainly focused on oral drug delivery, where PLGA was the most frequently used biopolymer. One study focused on the encapsulation of a water solution of BSA using an outer solution of PLGA dissolved in either DCM or a combination of DCM and DMF [107]. The other work encapsulated ranibizumab, a protein drug used for the treatment of age-related macular degeneration, using PLGA dissolved in a combination of DCM and acetonitrile as the outer solution [108]. Regarding the use of solvents, six of eight works used organic solvents, mainly for the outer feed. This is because the use of two immiscible solutions provides better core–shell separation by minimizing interdiffusion between layers [109]. A solution of ethyl acetate and n-butanol, along with acetylated dextran as carrier, was used as the outer feed for the encapsulation of anthrax protective antigens dissolved in the inner water solution [110]. Rasekh et al. [94] coaxially electrosprayed angiotensin II using NOSC as carrier for the inner solution and tristearin dissolved in DCM as outer solution. Since the literature found was focused on the production of oral delivered drugs, it would be necessary to take into consideration the need to apply two completely immiscible food-grade solvents to produce encapsulates oriented for food fortification.

Voltages applied ranged from 5 to 22.5 kV, similar to the values used for monoaxial electrospraying (2.67–20 kV). The effect of voltage was studied for the encapsulation of angiotensin II using tristearin and NOSC as carriers inner and outer carriers, respectively [94]. The applied voltage values were 20 and 30 kV, and the authors compared the stability of the enzyme using an ELISA, finding that at 30 kV the concentration of angiotensin II in the microparticles was reduced by approximately 20%. For the encapsulation of alkaline phosphatase with PEO as outer carrier [95], the voltage was optimized to 22.5 kV. Similarly, these authors found that this high voltage resulted in a loss of activity of the enzyme up to 40% compared to the activity obtained by monoaxial electrospraying at 15.5 kV.

Other parameters affecting particle characteristics are feed flow rates (inner and outer) and nozzle diameters. For the inner solutions (core), feed flow rates of 0.02–3.6 mL/h were used, while for the outer solutions (shell), 0.1–18 mL/h was used. Regarding the nozzle diameters, they ranged from 184 to 1000 μm for inner capillary and 603 to 2000 μm for the outer capillary. As previously mentioned in the previous section, increasing feed flow rate and nozzle diameters typically results in larger particles. This agreed with the data obtained by Zhao et al. [112], where alkaline phosphatase was encapsulated using CMC as inner carrier and alginate and PEGDA as outer carriers. They reported the highest feed flow rates (1.8 mL/h for the core and 3.96 mL/h for the shell) in the literature and obtained the largest particles at 440 μm. However, the opposite conclusion was obtained after comparing the encapsulation of angiotensin II (using NOSC as inner carrier and tristearin outer carriers) [94] and the encapsulation of alkaline phosphatase with PEO as carrier [95]. Both studies used similar nozzle diameters (1000 μm (inner)–2000 μm (outer), and 900 μm (inner)–1900 μm (outer), respectively), but the first study used feed flow rates 10 times higher. Even though larger particles would be expected for the angiotensin II encapsulation, due to the higher flow rates, their size was up to 86% smaller. In fact, they obtained the smallest particles, which could be due to the nozzle–collector distance, the highest reported in the literature at 20 cm, and the slightly higher voltage used. Coaxial electrospraying of bovine hemoglobin also resulted in small particles of 0.37 μm, as it was particularly important to obtain nano/microcapsules in the range of 0.1 to 3 μm to effectively avoid extravasation through the blood vessel wall and act as oxygen carriers [111].

High EE values were obtained for all the studies reported in the literature (Table 3), ranging from 70% to 99%. These values are higher than the ones obtained for monoaxial electrospraying, where four of the nine reported EE values were under 50%. Zamani et al. [107] reported ranges of EE from 46.7 ± 4.3% to 74.6 ± 2.9%, which were linked to incomplete encapsulation due to inner feed flow rates being too high as well as high concentrations of BSA in the core. The highest EE found was obtained for the encapsulation of alkaline phosphatase with PEO as outer carrier [95]. They also compared the effect of monoaxial and coaxial electrospraying, confirming that the EE was increased in core–shell structures.

Although coaxial electrospraying has exhibited promising outcomes, the encapsulation of bioactive protein hydrolysates or peptides has only been minimally investigated. Thus, further studies are required to fully evaluate the feasibility of this technology for the encapsulation of bioactive peptides. Particularly, there is a need to investigate the use of food-grade solvents and to optimize processing conditions that lead to encapsulates with potential use in food fortification.

## 5. Activity Retention and Release of the Encapsulated Protein-Based Bioactives

Spray-drying and electrospraying techniques are viable encapsulation methods of protein-based bioactives. After encapsulation, it is fundamental that these bioactive compounds maintain their original activity. Moreover, they should remain active until reaching the target organ, where they will exert their activity [114]. However, research studying the factors that affect the preservation of activity and release of encapsulated protein-based bioactives is scarce.

Maintaining activity after encapsulation is one of the most important challenges facing protein delivery, as this can be limited by protein aggregation or denaturation in the particles [115]. Regarding the works in the literature studying the retention of activity, it is striking that 7 out of 12 deal with antioxidant activity (i.e., DPPH free radical scavenging activity, ferric reducing antioxidant power, metal-chelating activity). This trend might be due to the increasing focus on the use of natural antioxidants as both bioactives in humans and functional ingredients in food products to avoid lipid oxidation [116,117,118]. Fish collagen hydrolysates exhibiting 2,2-diphenyl-1-picrylhydrazyl (DPPH)-inhibitory activity were spray-dried using MD as carrier at different ratios. Antioxidant activity was lost when MD was used as carrier, with a decrease in activity from 78.36 ± 12.29% when free collagen was spray-dried to 33.59 ± 6.47% when 80% of MD with 10–12 dextrose equivalent was used [34]. Other studies spray-dried fish hydrolysates with MD and GA, reporting that the presence of carbohydrates from the carriers decreased antioxidant activity [52]. High retention of activity (>60%) was found by two studies analyzing the antioxidant activity of spray-dried flaxseed protein hydrolysates with MD by different methods (i.e., DPPH free radical scavenging, ABTS free radical scavenging, hydroxyl radical scavenging, reducing power, nitric oxide scavenging) [36,37]. On the other hand, spray-drying of oyster protein hydrolysate included in emulsions, with MD as carrier and cholesterol for the oily phase, resulted in reduced free radical scavenging activity [41]. This was related to the high inlet temperature (170 °C) altering the lipid membrane and partially degrading the bioactive compound.

The activity retention capacity of different encapsulated enzymes by electrospraying was also measured. Alkaline phosphatase with PEO was encapsulated both by monoaxial and coaxial electrospraying, retaining ~100% activity when monoaxially electrosprayed. However, when coaxial electrospraying was applied, only 60% of the activity was retained, which was attributed to the high voltage (22.5 kV) applied compared with monoaxial spraying (15.5 kV) [95]. Amylase included in emulsions using dextran and sodium alginate as carriers was encapsulated by electrospraying; however, the high activity observed for amylase (96%) was related to a low EE, indicating that the substrate had better access to the enzyme due to the location of the enzyme at the surface of the particle [102].

In addition to the efficient encapsulation of protein-based bioactives, it is essential for the application of these methods that these compounds can be released from the carrier matrix while maintaining their activity. Despite the importance of studying these parameters, literature on release kinetics or tailored release approaches for the encapsulation of protein-based bioactives is limited. In general, the release of nano/microencapsulated bioactive compounds occurs in three stages: i) surface release, which may be caused by inadequate entrapment (low EE) in the carrier matrix, ii) diffusion via swelling of the carrier matrix, and iii) erosion of the carrier matrix [119]. It is usually desired to avoid burst release as much as possible. For that, it is necessary to take into consideration the characteristics of the carrier, which should not totally solubilize in the release medium, not interact with protein-based bioactives, and be protective against external factors, such as acid pH during gastric digestion [120]. The most common method to measure protein release from the delivery systems found in the literature is by agitation in PBS release buffer. Bock et al. [92] studied the release during 81 days of SA electrosprayed using PEG as carrier. The initial burst release was dependent on the protein load, PEG load and PEG molecular weight (MW). Higher SA load resulted in a strong burst release, up to 60% in the first 24 h. A burst-free release of the SA with sustained release up to 84 days was achieved combining low protein loading (1%) and low MW PEG (6 kDA). These results agreed with the data obtained for the electrospraying of BSA emulsions using PLGA as the carrier [99]. In order to compare the release profile obtained by coaxial electrospraying versus emulsion monoaxial electrospraying, Zamani et al. encapsulated BSA using PLGA as carrier [107], finding that although coaxial electrospraying enhanced the EE, it also resulted in a stronger burst release (24–27%) than emulsion electrospraying (8–12%). This was due to the centralized distribution of the bioactive in the coaxially produced encapsulates allowing fast release once the solvent reached the bioactive, contrary to the compartmentalized distribution of the bioactive in the encapsulates produced by emulsion electrospraying (Figure 4). Although this method does not take into consideration the gastrointestinal conditions to which the capsules would be subjected during digestion, it allows us to estimate the stability of the capsules.

## 6. Bioaccessibility of Encapsulated Protein-Based Bioactives and Enrichment of Food Matrices

Bioactive peptides and proteins must be bioaccessible for their potential use in oral delivery systems for the development of supplements and functional foods. Bioaccessibility refers to the fraction of the biocomponent that, after digestion, becomes accessible for absorption through the epithelial layer of the gastrointestinal tract [121]. Bioaccessibility can be determined with good results using in vitro methods that simulate the biochemical and mechanical conditions of the gastrointestinal digestion.

The effect of gastrointestinal digestion of spray-dried nanoliposomes of red tilapia viscera hydrolysates over antioxidant and ACE-inhibitory activities were studied [43]. Gastric digestion with pepsin induced degradation of the peptides, resulting in loss of antioxidant activity, while increasing ACE-inhibitory activity. For both bioactivities, the intestinal digestion resulted in enhanced inhibition, up to 10% compared to the original hydrolysates. This was attributed to the release of new oligopeptides after digestion with pancreatin, as bile salts in the intestinal phase promoted swelling and disruption of the vesicles, thus leading to leakage of bioactive peptides. Spray-dried peptides derived from spent brewer’s grain were encapsulated with locust bean gum, *P. columbina* phycocolloids, or DM as carriers, and their bioaccessibility was measured by analysis of the size distribution of the peptides and the retention of their ACE-inhibitory activity [48]. They found that carrier selection affected greatly to the protection of the peptides during digestion, achieving highest protection when P. columbine phycocolloids were used as wall material. Regarding ACE-inhibitory activity, encapsulated peptides showed higher activity than free peptides after digestion, demonstrating that partial protection of bioactive peptides against digestive enzymes was possible. Similar results were obtained by spray-drying *P. lunatus* hydrolysates encapsulated by spray-drying with MD and GA as carriers [42], where the ACE-inhibitory activity, as well the DPP-IV and α-amylase activity, of free hydrolysates was severely affected by simulated gastrointestinal digestion (IC50 ≈ 300 µg/mL) compared to the retained activity of encapsulated hydrolysates (IC50 < 200 µg/mL). Coaxial electrospraying of alkaline phosphatase, with CMC as core carrier and a mixture of alginate and poly(ethylene glycol) diacrylate (PEGDA) as a carrier in the shell, was able to protect the enzyme from potential degradation during simulated gastrointestinal digestion [112].

Gómez-Mascaraque et al. [49] evaluated the changes in the profile of peptides obtained from whey protein hydrolysate, which was spray-dried using gelatin and CS as encapsulating agents, after digestion. After comparing the chromatograms of the original hydrolysate and the digested capsules, they found that simulated digestion of the free hydrolysate resulted in a “remarkable change” in the identified peptides, compromising bioaccessibility, whereas digestion of CS microcapsules retained the highest number of identified peptides. The higher digestion of the hydrolysate-loaded gelatin capsules was linked to the proteinaceous origin of the carrier, possibly also digested during the assay. Another work studied the release kinetics of bioactive compounds during and after in vitro digestion by measuring changes in absorbance of spray-dried rapeseed peptides [57]. This study found that microparticles did not produce an initial burst during gastric digestion, but rather a slow release of encapsulated peptides during the intestinal stage, which would increase bioaccessibility. Paz-Samaniego et al. [113] performed a simulation of gastrointestinal digestion with a complex Simulator of the Human Gastrointestinal Tract (Simgi). Insulin-loaded microcapsules obtained by coaxial electrospraying with maize bran arabinoxylans (core) and maize wastewater arabinoxylans (shell) as encapsulating agents were passed through five different reactors, simulating the stomach, small intestine, and the three regions of the colon: ascending, transverse, and descending. In this way, they found that 76% of the encapsulated insulin reached the colon without being degraded in the stomach.

In addition to potential degradation during gastrointestinal digestion, protein-based biocompounds can easily react with complex food matrices, leading to alterations in their bioactivity. These matrices, such as soups or baked goods, can undergo thermal and high-pressure conditions that would make it difficult to maintain peptide stability [122]. Thus, encapsulation of bioactive peptides and proteins could potentially benefit their stability when incorporated into food matrices. Only three studies regarding the inclusion in food matrices of protein-based bioactives encapsulated by spray-drying or electrospraying were found in the literature. Yogurt was fortified with spray-dried weakfish hydrolysates exerting antioxidant and ACE-inhibitory activities using MD as carrier [48]. After a week of storage, not only were antioxidant and ACE-inhibitory activities maintained, but greater rheological stability was provided by the encapsulated hydrolysates. Whey protein hydrolysate-loaded gelatin or CS capsules were used to enrich yogurt produced by lactic acid fermentation [49]. During the fermentation process, a large part of the peptides present in the hydrolysate was lost due to susceptibility to the living starter cultures. After fermentation, the peptide profile of the yogurt enriched with free hydrolysate, encapsulation with CS or encapsulation with gelatin was analyzed. Enrichment with free hydrolysates resulted in the protection of 30 of the 58 peptides initially identified. The same amount was protected in hydrolysate-loaded CS capsules, but five different peptides were found. When hydrolysates were encapsulated with gelatin, only 21 peptides were protected; however, it is difficult to determine with certainty the protective effect of gelatin, since its proteinaceous nature resulted in very complex chromatograms that did not allow conclusive conclusions. Spray-dried hydrolysates from pink perch meat were used to fortify a sweet-corn vegetable soup [52]. Both particles with and without encapsulating agents (MD and GA) were used, and although higher activity was retained when no carrier was used, their use improved sensory acceptability. The activity loss on MD/GA hydrolysate particles was linked to interaction between the encapsulating agents and the hydrolysates.

## 7. Conclusions and Future Perspectives

Bioactive peptides and protein hydrolysates are interesting ingredients for the production of functional foods and nutraceuticals due to their high bioactive potential and nutritional value. However, their physicochemical properties (e.g., bitter taste) and potential degradation during digestion have been shown to hinder their use. This work addresses the application of encapsulation technologies such as spray-drying and electrospraying for encapsulation, protection, and release of bioactive peptides and protein hydrolysates. For each encapsulation technology, both monoaxial and coaxial configurations were considered. Various parameters that may affect particle morphology and encapsulation efficiency (e.g., formulation processing method or carrier) were investigated, as well as the specific process parameters for both technologies (e.g., inlet and outlet temperature of the drying air, electrical potential, feed flow, injector–collector distance). It is worth noting that while there has been a significant effort to produce new bioactive peptide sequences, there has been a lack of attention towards their stabilization. Additionally, research on encapsulating protein-based bioactives using monoaxial spray-drying is scarce, and practically nonexistent for coaxial spray-drying. Although some research has been conducted on the encapsulation of bioactive peptides by both monoaxial and coaxial electrospraying, it has mainly been focused on pharmaceutical applications and parenteral supplementation. As a result, there is a significant gap in research regarding food application and oral supplementation. Altogether, encapsulation has an important role in maintaining the efficacy of functional foods containing bioactive peptides and proteins. However, the current widely used spray-drying method has only been studied in monoaxial configuration and the potential of the coaxial mode remains to be investigated. Although advanced methods such as electrospraying encapsulation have shown promise in pharmaceutical development, there is a need to explore their applications in the food industry. As research in this field continues, we can expect to see advances in the nano/microencapsulation of bioactive peptides for their application in functional foods.

Finally, works focusing on release studies, retention of activity, and bioaccessibility are limited, especially regarding the study of monoaxial electrospraying. Overall, there is a lack of research concerning the bioaccessibility of protein-based bioactives encapsulated by spray-drying or electrospraying, as well as their stability in food matrices. To the authors’ knowledge, no work has been directed towards the bioaccessibility analysis of monoaxial electrospraying of protein-based bioactives, nor has any study yet compared the effect of monoaxial versus coaxial electrospraying of these biocompounds in their bioaccessibility or use for food matrix enrichment. Hence, future research should prioritize two aspects for activity retention of bioactive peptides: (i) exploring the possibilities of coaxial encapsulation techniques that can improve the entrapment of bioactive peptides, and (ii) investigating the effects of nano/microencapsulation on the stability and release of peptides in the gastrointestinal environment to enhance bioavailability.

## Figures and Tables

**Table 1:** Studies on the encapsulation of protein-based bioactives by monoaxial spray-drying.

	Formulation						Process Variables					Capsule Characteristics		Ref.
	Hydrolysate/Peptide	Bioactivity	Carrier	Solvent	Peptide: Carrier Ratio (*w*/*w*)	Prep. Method	T Inlet (°C)	T Outlet (°C)	Drying Air Flow Rate (L/h)	Feed Flow Rate (mL/h)	Nozzle Diam. (μm)	Size (μm)	EE (%)	
Hydrolysate	Buffalo whey proteins hydrolysate	–	GA and MD	Water	1:10 and 1:15	Blend	105	90	9000	160	700	2.0/20.0	>95%	[31]
	Collagen hydrolysate	Organ formation	GA and MD	Water	1:4	Blend	150–170	50–60	–	–	–	0.4–50	86 ± 7–85 ± 4%	[32]
	Edible bird’s nest hydrolysate	Antioxidant	MD/MD and CMC/MD and XG	Water	1:3.33	Blend	180 ± 2	90 ± 2	–	600	700	–	85.97 (MD) 81.15 (MD + CMC) 86.58 (MD + XG)	[33]
	Fish collagen hydrolysate	Antioxidant	MD	Water	1:9 and 1:4	Blend	140 ± 1	80 ± 0.5	–	–	700	<425 µm	–	[34]
	Fish protein hydrolysates	Antioxidant	MD	Imidazole acetate buffer	1:200	Blend	180	90	–	500	500	61.5 ± 1.7–183 ± 2.8	–	[35]
	Flaxseed peptide fractions	Antioxidant	MD	Water	1:3	Blend	110 ± 1	60 ± 2	540	300	500	9–11	–	[36]
	Flaxseed protein hydrolysate	Antioxidant	MD	Water	1:1, 2:1 and 3:1	Blend	130 ± 1	75 ± 2	–	300	700	~ 5	–	[37]
	Flaxseed protein hydrolysate	Antioxidant	MD	Water	1:1	Nanoliposome	130 ± 1	75 ± 2	–	300	500	0.326–0.353	72.12–84.99	[38]
	Flaxseed protein hydrolysates	Antioxidant	MD	Water	1:1 (*v*/*v*)	Nanoliposome	130 ± 1	73 ± 2	540	300	500	0.132 ± 0.015–0.86 ± 0.012	84.0 ± 1.9–90.7 ± 1.6	[39]
	Mushroom protein hydrolysate	Food flavoring	GA	Water	1:2	Blend	150	95	–	300 g/h	500	–	–	[40]
	Oyster protein hydrolysate	Antioxidant	MD	Water	1:1	Nanoliposome	170	–	–	300	–	0.392–0.719	71.43–82.36	[41]
	*P. lunatus* hydrolysate	Antidiabetic	MD and GA	Water	1:25 and 1:10	Blend	160 ± 4	88 ± 2	33.6	1380	–	3.3–6.8	59.9–82.0	[42]
	Red tilapia viscera hydrolysate	Antioxidant and antihypertensive	Soy rapeseed lecithin	Phosphate buffer	1:5	Nanoliposome	130	62	–	630.5	700	0.25–0.31	80–81	[43]
	Watermelon seed hydrolyzed protein	antioxidant	MD and sucrose	Water	–	Blend	150–180	80	–	200	–	10–12.9	–	[44]
	Soy protein hydrolysates	Antioxidant	Soy protein isolate and MD	Water	1:1.2 and 1:0.8	Blend	180	80–90	–	900	700	–	–	[45]
	*Spirulina platensis* hydrolysate	Hepatoprotective, antioxidant, anticancer, etc.	Mannitol and CS	Water (mannitol) and acidified water (CS)	1:10 (*w*/*w*)	Blend	120	70	500/600	300	500	14.24 ± 2.66	~ 100	[46]
	*Spirulina platensis* hydrolysate	Antioxidant	MD and CS	Water and acetic acid	60:40	Nanoliposome	130	75	–	–	–	1–3	88.0–89.0	[47]
	Stripped weakfish hydrolysate	Antioxidant and ACE inhibitor	MD	Water	60:40	Blend	130	70 ± 2	–	–	–	–	–	[48]
	Whey protein hydrolysate	–	CS/gelatin	Acetic acid 20%	3:1 (gelatin) 15:1 (CS)	Blend	90	50 ± 5	9000	–	–	0.603 ± 0.627 (G) 0.571 ± 0.440 (C)	–	[49]
	Whey protein hydrolysate	Physiological functionality	MD/MD and β-CD	Water	30:70	Blend	200	90 ± 5	-	1000	–	2.47–3.26	–	[50]
	Whey protein hydrolysate	Infant formulas	MD	HOSO (O) Water (W)	1:2 and 1:4	O/W emulsion	170	95 ± 3	38000	360	700	10	84.8–97.2	[51]
	Pink perch meat protein hydrolysate	Antioxidant	None/MD and GA	Water		Blend	160	80	–	900–1200	500	4.05–17.3 (no carrier) 5.12–15 (with carrier)	–	[52]
Peptide	Brewers’ spent grain digested peptides	ACE-I inhibitor	LBG and MD/LBG, PG and MD/PG and MD	Water	1:6	Blend	180 ± 2	96 ± 8	357	180	700	5–7	>90%	[53]
	Exenatide	Antidiabetic	Mannitol and PLGA	DCM and DMC (O) and water (W)	20:1 (*v*/*v*)	W/O emulsion	60	–	–	180	–	4.83 ± 1.79	84.65 ± 2.93	[54]
	Glutathione	Antioxidant	CS/β-CD	Water (β-CD) Acetic acid and water (CS)	1:3.5 (β-CD) 1:2.5 (CS)	Blend	200 (β-C) 130 (C)	72 (β-C) 47 (C)	–	600	–	(β-CD < chitosan)	62.4 (β-CD) 25 (CS)	[55]
	Nisin	Antimicrobial	Pectin/alginate	Water (Blend)/Soybean oil (O) and water (W1, W2)	1:2.5 (pectin) 1:125 (alginate)	Blend/W1/O/W2 emulsion	140 ± 5	70 ± 5	–	900	700	17.91–18.67 (B) 44.87–66.59 (E)	63.70 ± 1.31–69.88 ± 1.10 (B) 72.80 ± 1.98–84.66 ± 1.20 (E)	[56]
	Rapeseed peptides	Dietary protein source	Rapeseed protein isolates	Water	1:2	Blend	135 ± 2	74 ± 2	450	350	–	6.2 ± 0.12–8.5 ± 0.21	87.1 ± 1.2–94.7 ± 1.8	[57]
	Rapeseed peptides	Dietary protein source	Rapeseed protein isolates	Water	1:1, 1:2 y 2:1	Blend	135 ± 2	74 ± 2	450	350	–	5.8 ± 0.01–16.3 ± 0.12	60 ± 0.9–72 ± 1.1	[58]
	Soy peptides	Antihypertensive	Modified starch and MD (W2)	MCT oil (O) and water (W1, W2)	1:1 (*w*/*w*)	W/O/W emulsion	150	–	–	900–1200	–	1.44 ± 0.04–8.39 ± 0.21	29.51 ± 0.89–45.83 ± 0.47	[59]
Protein	Ovalbumin (OVA)	Vaccine antigen	Dextran sulfate, mannitol and PLARG	Water	1:9	Blend	120	–	–	60–600	700	1–10	99–110	[60]
	Capreomycin oleate	Antituberculosis	HA and DPPC	Water (DPPC) Ethanol (HA)	75:25 (*v*/*v*)	Blend	110	60–65	500	1020	700	2.06–9.14	56	[61]

T inlet (°C): inlet temperature of the drying air in °C; T outlet (°C): outlet temperature of the drying air in °C; EE (%): encapsulation efficiency in %; nozzle diam.: nozzle diameter; O/W: oil-in-water; W/O: water-in-oil; GA: gum arabic; MD: maltodextrin; CMC: carboxymethyl cellulose; XG: xanthan gum; CS: chitosan; β-CD: β-cyclodextrin; HOSO: high oleic sunflower oil; LBG: locust bean gum; PG: *P. columbina* phycocolloids; PLGA: poly(lactic-co-glycolic acid); PLARG: poly-l-arginine; DPPC: 1,2-dipalmitoyl-sn-glycero-3-phosphocholine; HA: hyaluronic acid.

**Table 2:** Studies on the encapsulation of protein-based bioactives by monoaxial electrospraying.

	Formulation						Process Variables				Capsule Characteristics		Ref.
	Hydrolysate/Peptide	Bioactivity	Carrier	Solvent	Peptide: Carrier Ratio	Prep. Method	Voltage (kV)	Feed Flow Rate (mL/h)	Distance N-C (cm)	Nozzle Diam. (μm)	Size (μm)	EE (%)	
Peptide	Alpha-calcitonin gene-related peptide	Vasodilator	Alginate	Water	1:2 (*w*/*v*)	Blend	6	60	7 × 10^−3^	–	194.23 ± 10.08	–	[97]
	Peptide pp65489-503 CMV 4–peptide mix	Immune stimulatory	PLGA	TFE/DMSO	1:22 (*w*/*w*)	Blend	10	0.01	14	30	200	84–85	[98]
Protein	SA	–	PCL and PEG/PLGA and PEG	Chloroform (PCL and PEG)/DCM (PLGA and PEG)	10:90, 5:95, 15:85	Blend	10	0.5–3	15 or 25	450–800	5.6 ± 0.8 (PLGA) 12.0 ± 4.0 (PCL)	20–40	[92]
	BSA	–	PLGA	Acetone (PLGA) Ethanol and acetic acid (BSA)	1:1, 1:4, 1:9	Blend	10, 15 and 20	36 × 10^−3^ and 72 × 10^−3^	10, 15 and 20	–	0.085–0.26	–	[93]
	BSA	–	PLGA	Chloroform (O) and water (W)	–	W/O emulsion	6	1	20	–	9.6 ± 1.4–7.2 ± 2.4	92–80	[99]
	BSA or porcine interleukin-1β	Vaccine adjuvant	Alginate	Water	2:1, 4:1, 6:1	Blend	12	0.1	20	511	20	50 (BSA)	[100]
	Bone morphogenetic protein 2	Bone regeneration	PLGA, BSA	DMF (PLGA) and water (BSA)	1:100 (*v*/*v*)	Blend	9–12	0.5	30	900	1.0 ± 0.6	39	[101]
Enzyme	Alkaline phosphatase	Enzyme- anti-inflammatory	PEO	Ethanol and water	1:7 (*v*/*v*)	Blend	9–15	0.5–1	12–22.5	610	0.73 ± 0.16	85.0 ± 4.0	[95]
	Amylase	Enzyme	Dextran and sodium alginate	Water	–	Water-in-water (*w*/*w*) emulsion	2.67–2.85	0.5	1 × 10^−3^	40–320	<1000	47 ± 3	[102]
	Streptokinase	Thrombotic disease treatment	PLGA	Water and DCM	1:100 (*v*/*v*)	Blend	13	0.1	10	180	0.037 ± 0.012	90	[96]
Hormone	Angiotensin II	Antihypertensive	NOSC	DMSO/water/DCM	1:1 (*w*/*w*)	Blend	15–19	1.08	0.1	508	1.057 ± 4 × 10^−3^	70–90	[94]

Distance N-C: distance between the nozzle and the collector; EE: encapsulation efficiency; Nozzle diam.: nozzle diameter; W/O: water-in-oil; SA: serum albumin; PCL: polycaprolactone; PEG: poly(ethylene glycol); PLGA: poly(lactic-co-glycolic acid); DCM: dichloromethane; BSA: bovine serum albumin; DMF: N,N-dimethylformamide; PEO: poly(ethylene oxide); NOSC: N-octyl-O-sulfate chitosan; DMSO: dimethyl sulfoxide.

**Table 3:** Studies on the encapsulation of protein-based bioactives by coaxial electrospraying.

	Formulation					Process Variables				Capsule Characteristics		Ref.
	Hydrolysate/Peptide	Bioactivity	Carrier	Solvent	Peptide: Carrier Ratio	Voltage (kV)	Feed Flow Rate (mL/h)	Distance N-C (cm)	Nozzle Diameter (μm)	Size (μm)	EE (%)	
Protein	Bovine serum albumin (BSA)	–	PLGA (outer)	Water (inner) DCM and DMF (outer)	–	9–11	0.1–0.2 (inner) 1–2 (outer)	–	300 (inner) 840 (outer)	5.4 ± 1.56–2.90 ± 0.76	46.7 ± 4.3–74.6 ± 2.9	[107]
	Bovine hemoglobin	Oxygen carrier	–	Ethanol	–	20	0.08 (inner) 0.48 (outer)	10	–	0.37	–	[111]
	Anthrax protective antigen	Antibacterial	Acetylated dextran (outer)	Ethanol, ethyl acetate and n-butanol	1:1.85	10–12	0.02 (inner) 0.85 (outer)	14	210 (inner) 603 (outer)	~1	–	[110]
	Ranibizumab	Age-related macular degeneration	PLGA (outer)	Water and EG (inner) DCM and acetonitrile (outer)	1:6	5	0.5 (inner)	16.95	–	1–2	70	[108]
Enzyme	Alkaline phosphatase	Detoxifying	CMC (inner) Alginate and PEGDA (outer)	Water	–	12.5	3.6 (inner) 18 (outer)	5	–	440	84	[112]
	Alkaline phosphatase	Anti-inflammatory	PEO (outer)	PBS (inner) Ethanol and water (outer)	1:2	22.5	0.02 (inner) 0.3 (outer)	15–20	1000 (inner) 2000 (outer)	1.29 ± 0.24	99	[95]
Hormone	Angiotensin II	Antihypertensive	NOSC and tristearin	Water (inner) DCM (outer)	1:1 (inner)	15–17.9	1.8 (inner) 3.96 (outer)	5–2.5	900 (inner) 1900 (outer)	0.17–0.26	92	[94]
	Insulin	Antidiabetic	MBA (inner) MWA (outer)	Water	4:1 (inner) 6.67:1 (outer)	16	1	5	184 (inner) 1194 (outer)	290	72	[113]

Distance N-C: distance between the nozzle and the collector; EE: encapsulation efficiency; PLGA: poly(lactic-co-glycolic acid); DCM: dichloromethane; DMF: N,N-dimethylformamide; EG: ethylene glycol; CMC: carboxymethyl cellulose; PEGDA: poly(ethylene glycol) diacrylate; PEO: poly(ethylene oxide); PBS: phosphate-buffered saline NOSC: N-octyl-O-sulfate chitosan; MBA: maize bran arabinoxylans; MWA: maize wastewater arabinoxylans.

## Data Availability

Not applicable.
